# Proportion and associated factors of glaucoma among outpatient department at university of Gondar comprehensive specialized hospital tertiary eye care and training center, northwest Ethiopia, 2021

**DOI:** 10.3389/fopht.2025.1521263

**Published:** 2025-03-05

**Authors:** Banchamelak Zeraye Yekunoamelak, Fisseha Admassu Ayele, Zinachew Mulat Bogale, Endalew Mulugeta Worku

**Affiliations:** ^1^ Department of Ophthalmology, School of Medicine, College of Medicine and Health Science, University of Gondar, Gondar, Ethiopia; ^2^ Department of Optometry, College of Medicine and Health Sciences, Comprehensive Specialized Hospital, University of Gondar, Gondar, Ethiopia

**Keywords:** proportion, glaucoma, outpatient department, Gondar, Ethiopia

## Abstract

**Purpose:**

The purpose of this study is to measure the proportion, types of glaucoma, and associated factors among outpatient departments at the University of Gondar Comprehensive Specialized Hospital Tertiary Eye Care and Training Center, Northwest Ethiopia.

**Methods:**

An institution-based cross-sectional study design was conducted on 934 participants who were selected by a simple random sampling method at entrance of tertiary eye care and training Center from September to November 2021. A structured questionnaire was used to collect the data through interviews, and the presence or absence, type, and stage of glaucoma were determined by reviewing the chart. The questionnaire was adapted from a previous study, and the data were entered into Epi Info version 7 and analyzed using SPSS version 20. Descriptive data were analyzed in terms of proportion, frequency, mean, and standard deviation. Binary logistic regression was utilized to identify determinant factors, with significance considered at a p-value less than 0.05.

**Results:**

A total of 934 study participants with an 85.33% response rate took part in the study. The mean age of the study participants was 55.67 SD ± 13.21 years. The proportion of glaucoma was 13.4% [(95% CI: (10.9, 15.8)], with Primary Open Angle glaucoma accounting for 96.3% of the total number of glaucoma cases. The age groups of 56-66 [AOR=3.80(95% CI: 1.99-7.26)], 67-87 [AOR=5.34(95% CI: 2.70-10.45)] and those who completed college or university [(AOR= 5.41(95% CI: 2.12-13.82)] were significantly associated with the presence of glaucoma.

**Conclusion:**

This study shows a high proportion of glaucoma compared to other studies, with Primary Open Angle Glaucoma being the most prevalent type. Increasing age and higher education level were significantly linked to the presence of glaucoma. Further research is needed to explore the relationship between education level and glaucoma.

## Introduction

Glaucoma is a progressive optic neuropathy characterized by thinning of the neuroretinal rim with excavation and an increase in the optic cup- to- disc ratio and deformation of the lamina cribrosa. Visual field loss is not detected in the early stage and visual acuity remains safe initially; however, its progression can lead to complete loss of vision (1).

Globally, glaucoma is the second leading cause of blindness, with about 3.6 million people becoming blind from glaucoma (2, 3). Primary open-angle glaucoma is high in Africa (2–4). The prevalence of glaucoma globally is increasing, with an estimated 76.0 million cases by the year 2020, and projected to raise to 111.8 million by 2040 (4, 5). It is much higher in Sub-Saharan Africa, where approximately half of glaucoma patients are unilaterally blind at their presentation to the hospital (6).

Many studies have shown that the prevalence rate of glaucoma increases with age (7–9). In Ethiopia, a national survey of low vision and blindness by 2005/6 showed that 5.2% (10), and in North shoa Merhabete woreda 27.7% of blindness is due to glaucoma (11).

Glaucoma decreases the quality of life, especially for elderly patients (12). Community screening is needed for early detection of the disease and treatment accordingly.

Even though a low vision survey was done in Gondar hospital, the real magnitude of glaucoma is hidden. This study aims to fill the gap about the proportion and type of glaucoma as well as the age-specific magnitude of glaucoma.

Glaucoma is a public health issue in Ethiopia but there are no sufficient reports about the magnitude of the disease, especially in the study area. Diagnosing glaucoma at the community level requires more instruments and experienced ophthalmic professionals. This study might provide insight into the magnitude of the disease and risk factors that contributing for the presence of glaucoma.

## Materials and methods

### Study design and setting

An institution-based cross-sectional study design was used to measure the proportion and associated factors of glaucoma among outpatient ophthalmic patients at the University of Gondar Comprehensive Specialized Hospital Tertiary Eye Care and Training Center, Northwest Ethiopia from September to November 2021.

#### Source population

All adult outpatients who visited the University of Gondar Comprehensive Specialized Hospital Tertiary Eye Care and Training Center.

#### Study population

All adult outpatients who attended the eye care center during the study period.

#### Inclusion criteria

All adult outpatients whose aged 40 years or older included in the study.

#### Exclusion criteria

Those unable to communicate, and mentally ill.

### Sample size determination

The sample size needed to assess the proportion rate of glaucoma was determined using the single population proportion formula on the following assumption. Level of significance (α) = 5% (with confidence level of 95%), Marginal error (w) = 2%, P=0.098 (hospital-based prevalence rate of glaucoma in Gondar 2020) (13), Z-value of 1.96 was used at 95% CI (n= sample size, P= proportion, w= marginal error).


$$\text{n =}\frac{{\text{z}}^{2}\text{α}/2\text{ p }(1-\text{p})}{{\text{w}}^{2}}=\frac{{\left(1.96\right)}^{2}(0.098)\;(0.902)}{{\left(0.02\right)}^{2}},\text{ n}=\;848.95$$

After adding 10% for non-response, the final sample size was calculated to be 934.

### Sampling technique

To select 934 patients, the average number of patients who attended ophthalmic services per day was 65, taken from the patient record book of the eye care service for the last month, and the estimated number of patients were seen per month were 1950. Thus K for this study was 2(k=N/n=1950/934≈2.25). The first participant was selected by simple random sampling, then every 2^nd^ patient was selected until the required sample size was achieved.

### Data collection tools and procedures

Data collection involved two components; patient medical chart review and a face-to-face interview using a structured questionnaire. The clinical characteristics including the diagnosis, type of glaucoma and its stage were obtained by reviewing the patient’s chart. The structured questionnaire includes socio-demographic characteristics and it is based on patient self-responses.

### Operational definitions

#### Glaucoma

Those patients who had glaucoma confirmed by MSc holder senior clinical optometrists and residents with fully investigated by using Volk lens, Gonioscope as well as FDT and the diagnosis is cross-check over by glaucoma specialist.

#### Primary open angle glaucoma

Patients those who have deep anterior chamber by slit lamp examination and have open angle by Gonioscope examination.

#### Primary open angle glaucoma

Patients those who have shallow anterior chamber by slit lamp examination and have closed angle by Gonioscope examination.

#### Stages of glaucoma

Early: Optic nerve abnormalities consistent with glaucoma but no visual field abnormalities and cup to disc ratio (CDR)< 0.65. Moderate: optic nerve abnormalities consistent with glaucoma and glaucomatous visual field abnormalities in one hemifield, and not within 5 degrees of fixation and moderate glaucomatous disc features of vertical CDR = 0.7–0.85. Advanced: patients who have CDR of 0.85 – 0.95 and glaucomatous visual field abnormalities in bug both hemifields, and/or loss within 5 degrees of fixation in at least one hemifield, and who can perceive light. Absolute: patients who have CDR of ≥ 0.95 and with a vision of no light perception (NLP) (14).

#### Visual acuity

It is used the ICD 12 definition of visual impairment. Mild/No visual impairment: 6/6 – 6/18, Moderate visual impairment: 6/18 – 6/60, Severe visual impairment 6/60 – 3/60, Blindness: < 3/60 (15).

### Data quality control

The questionnaire which was developed from different literature (14, 15) was translated into the local language, Amharic, and back- translated into English by experts and senior ophthalmologists. Pre-testing was done on 7% of the questionnaires out site the study area. Data collectors (ophthalmic nurses) received two days of training on the questionnaire, study purpose, and how to approach respondents. Daily supervision was conducted, and filled questionnaires were checked for completeness and consistency by the supervisor and principal investigator.

### Data processing and analysis

The data were entered to EPI INFO 7 and analyzed using SPSS version 20. Descriptive statistics such as proportion, percentage, mean and standard deviation were used to summarize the data. Bivariable and multivariable binary logistic regression were used to identify the factors associated with proportion of glaucoma. Model fitness was checked by using the Hosmer and Lemeshow goodness of fit test. A p-value of < 0.05 was considered statistically significant. The strength of association was assessed by using an adjusted odds ratio with a 95% confidence interval.

## Results

### Socio-demographic characteristics of study participants

A total of 934 study participants with an 85.33% response rate participated in the study. The mean age of participants was 55.67 SD ± 13.21 years. More than half of the participants were females 438(55.0%) and majority resided in urban areas 554(69.5%) (**Table 1**).

**Table 1:** Socio-demographic characteristics of the study participants among patient who were attending outpatient department in university of Gondar Comprehensive Specialized Hospital Tertiary Eye Care and Training Center, Northwest Ethiopia, 2021.

Variables	Frequency	Percentage
Age in year		
40 -55	413	51.8
56 -66	186	23.4
67- 87	198	24.8
Sex		
Male	359	45.0
Female	438	55.0
Residence		
Rural	243	30.5
Urban	554	69.5
Marital Status		
Married	482	60.5
Never Married	150	18.8
Widowed	100	12.5
Divorced	65	8.15
Educational Status		
No formal education	371	46.5
Primary School	130	16.4
Secondary School	135	16.9
College/University	161	20.2
Occupation		
Housewife	249	31.2
Government	167	21.0
Farmer	124	15.6
Private	172	21.5
Retired	85	10.7
Monthly income in USD		
< 16.7$	201	25.2
16.7-35.08$	245	30.8
35.1-87.7$	208	26.1
≥ 87.73$	143	17.9
Religion		
Orthodox	739	92.7
Muslim	48	6.0
Protestant	10	1.3

### Clinical characteristics of study participants

The proportion of glaucoma was 107(13.4%) with Primary Open-Angle Glaucoma (POAG) being the most common type 103(12.9%), zero primary angle closure glaucoma and 4(3.7%) secondary open and closed angle glaucoma. Nearly half (47.6%) of study participants had mild or no visual impairment, and 13.4% of visual impairment were caused glaucoma. Most participants 592(74.3%) had no systemic illness (**Table 2**).

**Table 2:** Clinical characteristics of the study participants among patients who were attending outpatient department at university of Gondar Comprehensive Specialized Hospital Tertiary Eye Care and Training center, Northwest Ethiopia, 2021.

Variables	Frequency	Percentage
Visual Acuity		
Mild/no visual impairment	379	47.6
Moderate visual impairment	264	33.1
Sever visual impairment	66	8.3
Blind	88	11.0
Patterns of ocular disease		
Glaucoma	107	13.4
Cataract	220	27.4
Retinal disease	44	5.5
Corneal disease	51	6.4
Refractive error	176	22.1
Blepharitis	48	6.0
Conjunctivitis	93	11.7
Others	60	7.5
Type of glaucoma		
No glaucoma	690	86.6
POAG	103	12.9
PACG	0	0
SOAG	2	0.25
SACG	2	0.25
Stage of glaucoma		
No glaucoma	690	86.6
Early	11	1.4
Moderate	26	3.2
Advanced	18	2.3
Absolute	52	6.5
Systemic illness		
Yes	205	25.7
No	592	74.3
Type of systemic illness	83	22.6
No	592	74.3
HPN	107	13.4
DM	34	4.3
Asthma	22	2.8
HIV	32	4.0
Others	10	1.3
Family history of glaucoma		
Yes	18	2.3
No	779	97.7
Previous ocular examination		
Yes	512	35.8
No	285	64.2

Others: (gastritis, urinary tract infection) HPN, hypertension; DM, diabetes mellitus; HIV, human immune deficiency virus; Others, (Pterygium, chalazion, pingicula). POAG, primary open angle glaucoma.

### Factors associated with the presence of glaucoma

The proportion of glaucoma was 107 (13.4%) (95% CI: 10.9 - 15.8%). POAG accounted for 103(12.9%). Participants in the age groups of 56-66 and 67-87 were three times and five times more likely to have glaucoma than those in the age group of 40-55. Participants who finished college or university were 5 times more likely to have glaucoma as compared to those who have not formal education (**Table 3**).

**Table 3:** factors associated with disease of glaucoma among outpatient department at university of Gondar Comprehensive Specialized Hospital Tertiary Eye Care and Training Center, Northwest Ethiopia, 2021.

Variables	No glaucoma	Glaucoma	COR (95%CI)	AOR (95%CI)	p-value
Age					0.000
40-55	385	28	1.00	1.00	
56-66	153	33	2.97 (1.73, 5.07)	3.80 (1.99,7.26)	0.000
67-87	152	46	4.16 (2.51, 6.90)	5.31 (2.70,10.45)	0.000
Sex					
Male	311	48	0.99 (0.66,1.49)		
Female	379	59	1.00		
Address					
Rural	203	40	1.43 (0.94,2.19)		
Urban	487	67	1.00		
Marital status					
Married	427	55	1.00		
Currently Single	263	52	1.54 (1.02, 2.31)		
Educational status			0.069		0.003
No formal education	310	61	1.00		
Primary school	118	12	0.52 (0.27, 0.99)	1.19 (0.55,2.62)	0.655
Secondary school	123	12	0.49 (0.26, 0,95)	1.77 (0.76,4.12)	0.185
College/university	139	22	0.80 (0.48,1.36)	5.41 (2.12,13.82)	0.000
Occupation					
Housewife	207	42	1.00		
Government	152	15	0.49 (0.26,0.91)		
Farmer	100	24	1.18 (0.68,2.06)		
Private	158	14	0.44 (0.23,0.83)		
Retired	73	12	0.81 (0.40,1.62)		
Average family income per month					
< 950	163	38	1.00		
951-2000	216	29	0.58 (0.34,0.97)		
2001-5000	182	26	0.61 (0.36,1.05)		
≥ 5001	129	14	0.47 (0.24,0.89)		
Type of systemic illness					
No	510	82	1.00		
HPN	88	19	1.34 (0.78,2.32)		
Others	92	6	0.41 (0.17,0.96)		

## Discussion

The study showed that the proportion of glaucoma was 107 (13.4%) (95% CI 10.9- 15.8) which is higher than in other studies conducted in Singapore and Brazil (3.4%) (14, 16), Cameroon (5.5%) (17) Nigeria (7.3%) (18), Kenya (4.3%) (19), in Ghana (8.5%) (20) in Jimma 10.24% (21), and in Gondar 9.79% (13). This difference might be due to the age of respondents and the fact that our institution is a tertiary eye care center. These factors might increase the magnitude of glaucoma cases compared to studies done in Debre Tabor 2.6% (22). This could be attributed to the large sample size of our study.

Regarding the type of glaucoma in this study, Primary- Open Glaucoma (POAG) accounts for 103(12.9%). It accounts 96.3% of the total glaucoma cases, which is almost similar to studies done in Ghana (9.43%) (20), and higher in Brazil (2.4%) (16), Cameroon (4.3%) (17), and Nigeria (6.2%) (18). It is lower than the studies conducted in Jimma 42.55% (21) and in Debre Tabor, 44.50% (22). The variation might be due to high sample size of our study and the fact that it is institution-based, which affects the age distribution of the result.

In consistent with several studies, as age increases, the risk of glaucoma increases (20, 21). Indian studies have shown that individual aged ≥70 years were 4% more likely to have glaucoma (7), while studies in Debre Tabor found that those over 60 year were 3 times more likely to have glaucoma (22). In this study, the age group of 56-66 was three times and 67-87 was five times more likely to have glaucoma than the age group of 40-55. Studies have also indicated that as age increases, intraocular pressure also increases, with thinner central corneal thickness and higher mean ocular perfusion pressure (23). Additionally, morphological studies have shown that outflow ability decreases with age, resulting in increased intraocular pressure due to the accumulation of extracellular materials in the trabecular meshwork (24).

Educational level was found to be significantly associated with the presence of glaucoma. Participants who completed their college or university (AOR=5.41(95% CI: 2.12, 13.82) were five times more likely to have glaucoma than those with no formal education. This could be because, individual with higher education may have more frequent eye examinations due to having awareness about the disease and a higher demand for vision and early detection of visual impairment. In this study 70% of participants who finished college or university had previous ocular examination compared to 59% of those with no formal education. In this study 81% of educated participants had no visual impairment compared to only 25% of illiterate participants. This may lead to an increased rate of glaucoma detection and further investigation may be needed in this area.

## Strength and limitation of the study

### Strength of the study

This study determined the true prevalence of glaucoma and identifies different the types of glaucoma along with their associated factors.

### Limitation of the study

The study not include pediatric population and was limited to hospital settings only, therefore it did no capture data on the community population.

## Conclusion

The study found higher proportion of glaucoma compared to other studies, with primary open-angle glaucoma (POAG) being the most common type. Increasing age and higher level of education were significantly associated with the prevalence of glaucoma. Further research is needed to explore the relationship between education level and glaucoma.

## Data Availability

Publicly available datasets were analyzed in this study. This data can be found here: The datasets used and/or analyzed during the current study are available from the corresponding author on reasonable request.
